# Rapid cognitive decline in a patient with chronic lymphocytic leukaemia: a case report

**DOI:** 10.1186/s13256-020-2360-9

**Published:** 2020-03-03

**Authors:** James Forryan, Jun Yong

**Affiliations:** 1 The Royal Liverpool and Broadgreen University Hospitals NHS Trust, Liverpool, UK; 2 Haematology Department, Duncan Building, Liverpool, UK

**Keywords:** Progressive multifocal leukoencephalopathy, Chronic lymphocytic leukaemia, Ofatumumab, Monoclonal antibodies, B-cell-depleting therapy

## Abstract

**Background:**

The use of monoclonal antibodies in various settings has been linked to the development of progressive multifocal leukoencephalopathy (PML). Whilst this association is well-described with agents such as rituximab and natalizumab, the literature describing the occurrence of PML with ofatumumab therapy (especially in a haematology setting) is sparse. This case aims to draw attention to the above association with a particular focus on the mechanisms by which B-cell-depleting therapy can precipitate PML during the treatment of haematological malignancy.

**Case presentation:**

A 68-year-old Caucasian man presented with acute-on-subacute confusion and reduced mobility. He had a history of chronic lymphocytic leukaemia for which he had completed six cycles of ofatumumab and chlorambucil 2 months prior to presentation. Biochemistry, physical examination and imaging were unremarkable on admission. Subsequent neurological examination demonstrated diminished reflexes and an extensor right plantar, while magnetic resonance imaging (MRI) assessment revealed white matter hyperintensities in the frontal lobes with restricted diffusion surrounding these areas. Cerebrospinal fluid (CSF) analysis demonstrated normal cell counts and chemistry but detected John Cunningham virus (JCV) via polymerase chain reaction (PCR), with a quantitative value of 41,850 gEg/ml. CSF immunophenotyping excluded malignant processes. A diagnosis of PML was confirmed, and with the support of palliative care, the patient was discharged to a hospice for ongoing care with the family’s agreement.

**Conclusion:**

PML remains a rare complication of ofatumumab treatment. Nevertheless, clinicians should maintain a certain level of suspicion for this risk, especially in the context of patients presenting with clinical syndromes of encephalopathy and focal neurologic deficits. Furthermore, research to better our understanding of the manifold links between B-cell function and JCV regulation could provide valuable information for use in the future prevention and treatment of PML.

## Background

Progressive multifocal leukoencephalopathy (PML) is a rare but increasingly common demyelinating disease of the central nervous system (CNS) with a particularly poor prognosis, most conspicuously so among patients with haematological malignancy (typically having a 90% mortality rate within 2 months of diagnosis) [1, 2].

The aetiologic agent, the JC virus (JCV), was first culture-isolated in 1971 and posthumously named after an early subject with PML, John Cunningham. JCV is of the *Polyomavirus* genus and is pervasive in healthy human populations, although pertinently, the JCV archetype (found in the urine of approximately 1/3 of adults) is not pathogenic [3, 4]. Therefore, whilst the archetypal form of the virus is attributed to the primary infection (usually occurring in childhood), for pathogenicity, the JCV requires explicit conditions. Studies have demonstrated homogeneity of the non-coding regulatory region (RR) in archetypal JCV, whilst the RR seen in isolated JCV prototypes from PML patients differs, with unique (to each individual) repeats of a 98-bp element that shows variation from the norm [5]. Therefore, rearrangement of the RR in the context of immunosuppression, rather than transmission, likely allows activation of the JCV. Furthermore, it is the RR that contains determinants of neurotropism and neurovirulence, giving further credence to its rearranging serving as a catalyst for PML [6].

The three most common PML populations comprise those with human immunodeficiency virus positive (HIV+) disease, haematological malignancies and relapsing-remitting multiple sclerosis (RRMS) on natalizumab [7]. Indeed, as human immunodeficiency virus (HIV) infection has become more prevalent throughout the world, and with the advent of B-cell-depleting immunomodulatory therapy in malignancy and autoimmune conditions, the prevalence of PML has risen; as an example, a 2018 study from Sweden looking at the incidence of PML per 100,000 found an increase in the average incidence rate from 0.026 (between 1988 and 2010) to 0.11 (between 2011 and 2013) [8].

Here we report a case of PML secondary to B-cell-depleting therapy with ofatumumab. Over the past decade, a new understanding of the role of B-cells in autoimmunity has begun to emerge. Whereas B-cells initially were thought to exert their effects predominately through antibody production, considerable evidence now exists to support their role in modifying T-cell function through cytokine production, co-stimulation and antigen presentation. Therefore, the wielding of B-cell depleting monoclonal antibodies in the treatment of autoimmune pathology and lymphoproliferative disorders should be appreciated for what it is—a double-edged sword with clear benefits of a more tolerable immune environment and suppression of uncontrolled lymphoproliferation—with the unwanted potential for previously suppressed viruses (i.e., JCV) to exploit the softening of immune regulation.

## Case presentation

A Caucasian male in his late 60s with a background of TP53-negative chronic lymphocytic leukaemia (CLL) presented through the Emergency Department with reduced mobility and confusion of 2 weeks’ duration. The diagnosis of CLL had been made 4 years prior and was originally staged as Binet A (less than three areas of lymphadenopathy and with no anaemia or thrombocytopaenia); unfortunately, the profound progression of symptoms and lymphadenopathy in the preceding year had necessitated a progression from surveillance to active management. The patient had therefore been enrolled in the RIALTO trial and completed six cycles of ofatumumab and chlorambucil 2 months prior to this presentation. Notably, a subtler deterioration in memory had occurred in the 2 months prior to admission, but before this, the patient was fully independent with normal cognition.

Other comorbidities included fatty liver changes, gout and recurrent respiratory tract infections associated with hypogammaglobulinaemia. A comprehensive medical history (including drug history) failed to explain his symptoms. No relevant family or travel history was present. On initial assessment in the Emergency Department, physical examination was unremarkable except for a Glasgow Coma Scale (GCS) score of 14/15 due to confusion. Neurological examination in the Emergency Department was documented as normal.

Routine bloodwork on admission (including full blood count, bone, renal and liver profile, thyroid function testing and vitamin assessments) was unrevealing. The white cell count was 6.6, with neutrophils of 4.8 and lymphocytes of 1.2. A chest X-ray and computed tomography (CT) brain demonstrated no acute lung or intracranial pathology, respectively. Microbiological sampling of the stool, urine and blood demonstrated no organisms on smear or culture. A CT neck/thorax/abdomen/pelvis showed interval improvement in the size of enlarged subcarinal, portocaval, right iliac and left iliac lymph nodes (compared to a previous CT of 8 months prior) and stable splenomegaly.

**Fig. 1:**
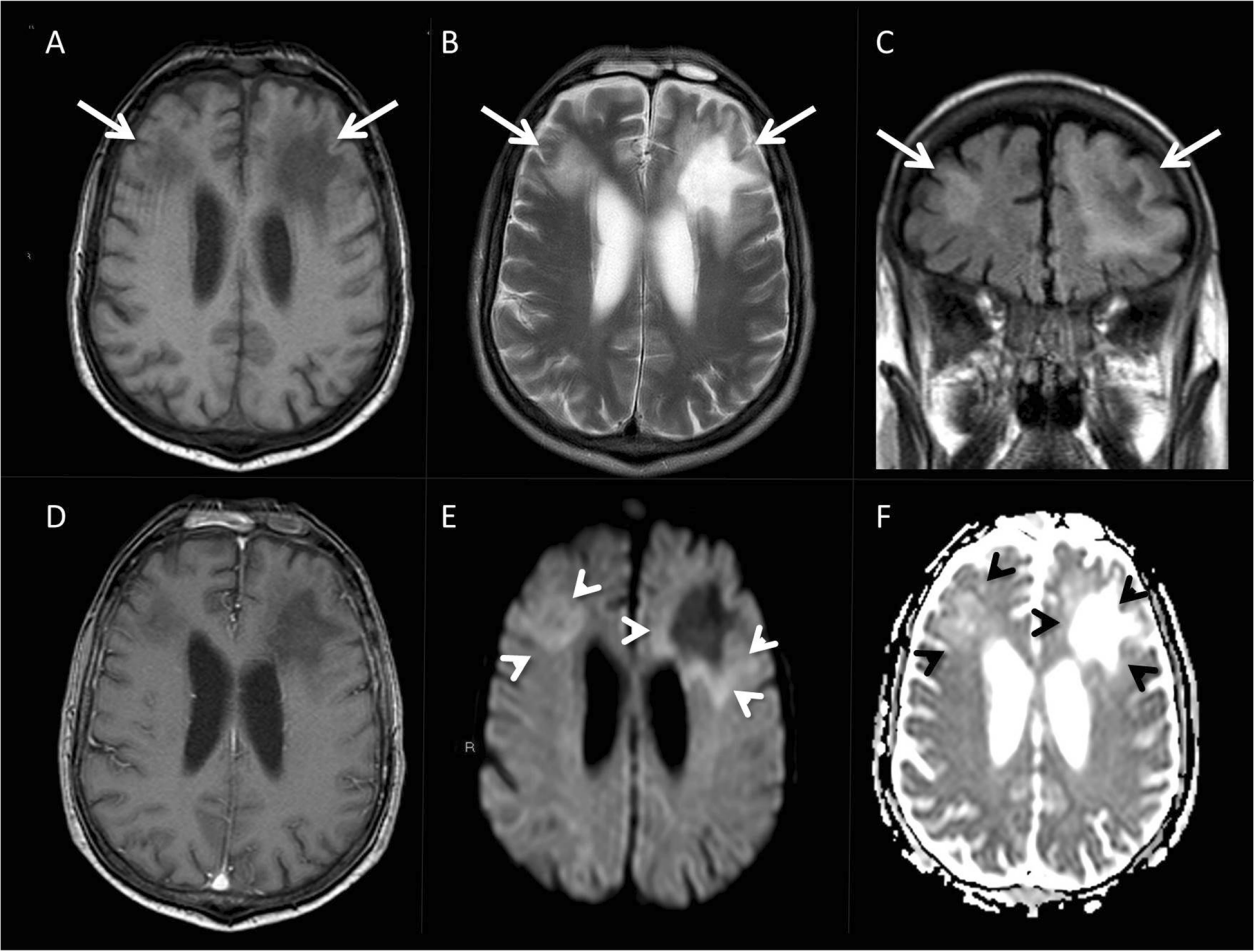
(**a**) Axial T1-weighted magnetic resonance imaging (MRI) showing asymmetric hypointense areas in the bilateral frontal white matter (arrows). (**b**, **c**) They appear hyperintense on the corresponding T2-weighted and FLAIR sequences. (**d**) The lesions do not enhance following intravenous contrast administration. (**e**, **f**) A thin rim of diffusion restriction can be seen along the advancing edge of the lesion on the DWI images and the corresponding ADC maps (arrowheads)

Early in the admission, concerns were raised by various members of the team about progressive cognitive impairment, loss of independence, inability to follow simple commands and a lack of insight. A further neurological examination was undertaken on admission to a medical ward which revealed diminished reflexes and an extensor right plantar response. A brain magnetic resonance imaging (MRI) (Fig. 1) then demonstrated white matter hyperintensities in the frontal lobes, with restricted diffusion in a flame front configuration surrounding the lesions. No abnormal enhancement was seen.

The MRI appearances, in conjunction with the clinical syndrome presenting itself, were felt to be highly suggestive of progressive multifocal leukoencephalopathy (PML). Other differentials, such as secondary central nervous system (CNS) lymphoma and CNS CLL infiltration, were considered less likely due to an absence of the typical avid enhancement usually seen on MR imaging. To help confirm a diagnosis, CSF analysis was undertaken. No organisms were grown from the CSF, and the white cell count was 44 per c.mm (100% lymphocytes). CSF protein was raised at 0.68. The CSF virology and opportunistic infection screen (cryptococcal, toxoplasma and *Mycobacterium tuberculosis*) were negative apart from detection of John Cunningham virus (JCV) via polymerase chain reaction (PCR), with a quantitative value of 41,850 gEg/ml; the symptomatology, radiological findings and CSF analysis were considered confirmatory of PML. The CSF immunophenotyping and morphology demonstrated no features of CLL or lymphoma. Ultimately, a brain biopsy was not performed because this would not have influenced the management, and the invasive nature of the procedure would have carried considerable risk given the patient’s performance status. We involved our palliative care colleagues in the care of this gentleman and were able to facilitate discharge to a hospice for ongoing symptom control and care. The patient sadly passed away roughly 3 months after the diagnosis of PML.

## Discussion

We report an adult patient with progressive cognitive decline and neurological dysfunction secondary to a diagnosis of PML. This occurred after B-cell-depleting therapy with ofatumumab in an attempt to treat his underlying CLL.

A tug of war has occurred in recent years between those who believe B-cells partake in both humoral and cellular immunity and those who dispute the role of B-cells in the latter. One can find evidence in the literature to support both arguments: for example, mice studies indicate that B-cell depletion impairs CD4+ T-cell memory and cytokine production, thus reducing protection against viruses [9], whilst others would argue the opposite, showing that cytotoxic T-cell memory is maintained even in B-cell-deficient mice subjected to lymphocytic choriomeningitis virus [10]. Nevertheless, studies that refute a role for B-cells in cellular immune responses have typically been published around the turn of the millennium, whereas they are now dwarfed in number by those supporting a role for B-cells in both types of immune response. Given the weight of evidence to support a modulatory role for B-cells in CD4 and CD8 T-cell responses, it is hard to ignore the association. Indeed, in a perversion of their intended action, B-cell depleting therapies have provided the milieu for researchers to shed light on the multi-mechanistic role that B-cells have in immune responses.

The existence of distinct B-cell populations within the subsets ‘effector’ (memory) and ‘regulatory’, and the ability of both types to modulate T-cell activity through antigen-presentation, cytokine release and co-stimulation, have long been known. Several studies have demonstrated a role for antigen-presenting B cells in the proliferation and differentiation of T-cells [11–13]. Furthermore, co-stimulatory B-cell surface molecules have their own role to play in T-cell responses; for example, a study demonstrated that in mouse bone marrow chimeras with CD80/86 knockout B-cells (but with other APCs retaining these CD markers), resistance to the induction of proteoglycan-induced arthritis was present. This suggested that CD80/86 upregulation on B-cells was a prerequisite for autoreactive T-cell activation and induction of arthritis; interestingly, proteoglycan-specific autoantibody titres were comparable in CD80/86 wild-type and knockout populations, thus offering more credence to the antibody-independent action of B-cells in cellular immunity [14]. The dichotomy between ‘effector’ and ‘regulatory’ B-cell is most apparent when related to their profile of cytokine production. Two effector subgroups, Be1 and Be2, produce cytokines linked to Th1 and Th2 responses respectively and are known to enhance T-cell-mediated immune responses. Conversely, B regulatory cells (the term Bregs was first coined by Mizoguchi and Bhan in 2002) also exist that produce an IL-10 predominant cytokine profile with T regulatory-cell (Treg) action, ultimately suppressing the T-cell response and creating a more tolerogenic immune environment.

Relevant to this particular case is the occurrence of PML in populations receiving B-cell-depleting therapy for lymphoproliferative disorders. The incidence of haematologic B-cell malignancies continues to rise in the Western world, but the advent of B-cell-depleting monoclonal antibody (mAb) therapy in the 1980s revolutionised treatment [15]. Identification of B-cell-restricted CD19 and CD20 antigens ushered in development of targeted mAb therapy, thereby leading to multi-mechanistic elimination of malignant B-cells [16]. Commonly used agents include rituximab, alemtuzumab and ofatumumab; the latter binds to a more proximal membrane epitope leading to improved complement-dependent cytotoxicity, with the potential caveat of increased susceptibility to the complications of B-cell depletion. As discussed, to say that decreased humoral activity explained the action of B-cell-depleting therapy would be an oversimplification; indeed, reviews and longitudinal case studies suggest a minimal role for specific antibody response as a regulatory factor in JCV infection [17, 18]. This supports suggestions that the role of B-cells in PML is multi-faceted, independent of antibody secretion and related to altered cell-mediated immunity; for example, B-cell-deficient IgM knockout mice lose their normal CD4 and CD8 response to a lymphocytic choriomeningitis virus (LCMV) variant, whilst non-IgM secretory B-cell-replete mice retained this function [19, 20]. Moving back toward the concept of Bregs, rituximab (an anti-CD20 monoclonal antibody) has repeatedly been shown to enrich the Breg pool, whilst depleting the Be1 levels and thus shifting the ratio of T- and B-cells in favour of the regulatory (Breg/Treg) phenotype [21]. Prior to B-cell-depleting therapy, the memory effector B- and T-cell activity predominates, and Be1/Th1 amplification loops are closely involved (when dysregulated) in the development of autoimmune disease [22]. This is where rituximab (and other B-cell depleting mAbs) come in—curtailing T-cell effector activity and depleting malignant B-cell clones in autoimmune and lymphoproliferative disease respectively. Nevertheless, where there is push, there is pull; shifting the immune environment to one of ‘tolerance’ and regulatory B- and T-cell activity removes the protection against JCV that the effector presence provided. One is left with the dilemma of how to retain the role of B-cells in JCV control—Th1-type cytokine release and propagation of Th1/CD8 activity—when treatment of these conditions necessitates B-cell depletion and leads to repopulation of the B-cell pool with naïve IL-10/35-producing Bregs.

The diagnosis of PML is contingent on the sum of clinical, radiological, virologic and histopathologic findings. The clinical signs are varied, the most common of which are motor impairment (typically hemiparesis), visual field defects, dysphasia and behavioural changes. MRI is the preferred imaging modality for detecting the changes synonymous with PML: multiple hyperintense (on T2-weighted and FLAIR images; hypointense on T1) white matter lesions within the affected lobes without oedema or mass effect, although a definitive diagnosis is only established by either virologic or histopathologic findings [23]. With well-established, evidence-based consensus guidelines recommending high-frequency MRI brain monitoring in high-risk natalizumab-treated multiple sclerosis patients, cases such as ours raise the question as to whether this practice should be extended to other at-risk groups [24]. Nevertheless, whilst studies in natalizumab-treated patients demonstrate improved survival and morbidity outcomes with early PML diagnosis [25, 26], similar results need to be demonstrated outside this group before sequential MR imaging is recommended more broadly.

For diagnosis, cerebrospinal fluid (CSF) examination for presence of the JCV is advised (ultrasensitive PCR techniques have a sensitivity > 95%), with several authorities accepting positive CSF JCV, clinical and radiological findings as confirmatory of PML [27]. Notably, a potential exists for both false-negative and false-positive CSF JCV DNA results (even with ultrasensitive PCR) [28]. In these instances, clinical assessment is paramount; proceeding to brain biopsy in high-suspicion patients with negative CSF tests is appropriate, while low CSF JCV titres in patients lacking clinical and imaging evidence of PML should be followed up with repeat testing and consideration of other pathology. Brain biopsy demonstrating asymmetric foci of demyelination and oligodendroglial cell intranuclear inclusions of JCV is an invasive, but extremely sensitive and specific, diagnostic tool.

As mentioned earlier, the prognosis of the disease is extremely poor. The incidence of PML is likely underestimated on account of its rapid progression and fickle presentation, thereby rendering diagnosis difficult. If confirmed, treatment options remain sparse (JCV is species-specific to humans, thus preventing animal model studies), with timely implementation of combination anti-retroviral treatment (cART) in HIV populations and cessation of immunomodulatory therapy being the only current options offering any survival benefit [29]. Currently, no specific prophylaxis for PML or effective anti-JCV treatment exists, and therefore, outcomes in PML are dependent on an individual’s ability to recover immune function [30]. Consequently, the poorest prognosis group is that of haematological malignancy; immune reconstitution is often not achievable in this group because of the innate bone marrow depression associated with the primary disease and long-term immune cell depletion that occurs secondary to treatment.

Moving forwards, the focus should be on prevention, early diagnosis and expanding treatment options. The identification of immunisation options is an ongoing endeavour; passive and active vaccines are at various stages of drug development, but isolated case reports with the use of JCV-directed cytotoxic T lymphocytes (CTLs) show promise [31]. Regular imaging and stratification of high-risk patients using expert-developed algorithms and JC biomarkers (e.g., the JCV antibody index) already play a role in the timely diagnosis of PML in certain patient cohorts and could be used more widely if studies demonstrated evidence in their favour [32]. Pharmacological treatment of established PML with antiviral agents preventing JCV cell entry, retrograde transport and DNA replication is in its infancy and currently is based on theoretical and anecdotal evidence [30]. Furthermore, as alluded to earlier, the understanding of B-cell function in JCV behaviour and PML is still unclear, although many studies suggest a significant role for B-cells in modifying the T-cell-mediated control of JCV infection. Therefore, further endeavours to better classify the relationships that exist between JCV and B-cells is likely to have a significant impact within the field of PML prevention and management.

## Conclusions

In conclusion, this case report aims to highlight the association between B-cell-depleting therapy and PML, particularly in the setting of haematological malignancy and with lesser used therapies such as ofatumumab. PML should take its place in any differential list for a patient receiving B-cell-depleting mAbs who presents with new neurology and cognitive defects as in our case. Additionally, we aim to bring attention to the mechanisms by which B-cell-depleting therapy can create an immune environment in which the JCV can become pathogenic; this raises the question as to how we can treat autoimmune and lymphoproliferative disease with B-cell-depleting therapy whilst mitigating the resultant increased risk of PML. Knowing the risk of PML with B-cell-depleting therapy, expediting research into JCV vaccination and pharmacological treatment, with concomitant development of evidence-based risk stratification algorithms and predictive biomarkers, seems the best way of reducing this risk at present. Finally, whilst rituximab’s effects on B-cell populations has been extensively studied (e.g., the shift from an effector B-cell pool to one made up of naïve B-cells and plasma cells), the same cannot be said of ofatumumab; clarification as to the phenotype of post-treatment B-cell populations with other B-cell-depleting mAbs could be revealing in terms of their risk-profile for PML compared to rituximab.

## Data Availability

Not applicable.

## Abbreviations

Breg: B regulatory cells
cART: combination anti-retroviral treatment
CLL: chronic lymphocytic leukaemia
CNS: central nervous system
CSF: cerebrospinal fluid
CT: computed tomography
CTLs: cytotoxic T lymphocytes
GCS: Glasgow Coma Scale (GCS)
JCV: John Cunningham virus
MRI: magnetic resonance imaging
PCR: polymerase chain reaction
PML: progressive multifocal leukoencephalopathy
RR: regulatory region
RRMS: relapsing-remitting multiple sclerosis
Treg: T regulatory-cell
